# Glycine Enhances Oxidative Stress Tolerance and Biocontrol Efficacy of *Sporidiobolus pararoseus* against *Aspergillus niger* Decay of Apples

**DOI:** 10.3390/foods12224121

**Published:** 2023-11-14

**Authors:** Jiaxi Wang, Qian Gao, Tianqi Fang, Yong Shen, Siyuan Jing, Na Guo

**Affiliations:** College of Food Science and Engineering, Jilin University, Changchun 130062, China; jiaxi21@mails.jlu.edu.cn (J.W.); gaoqian20@mails.jlu.edu.cn (Q.G.); fangtianqi0527@126.com (T.F.); shenyong19@mails.jlu.edu.cn (Y.S.); jingsy21@mails.jlu.edu.cn (S.J.)

**Keywords:** glycine, *Sporidiobolus pararoseus* CC-HS1, oxidative stress tolerance, biocontrol, apple

## Abstract

Apples are deeply loved by people because of their rich nutritional value, but they are susceptible to rotting. The use of antagonistic yeast is a promising method for controlling postharvest fruit diseases, but biocontrol efficacy of yeast will be weakened in environmental stress. In this study, the effects of glycine (Gly) on the oxidative stress tolerance and the biocontrol efficacy of *Sporidiobolus pararoseus* (*S. pararoseus*) against *Aspergillus niger* (*A. niger)* are discussed. Under the stimulation of H_2_O_2_, the yeast cells treated with Gly (1 mM) showed lower ROS content, less mitochondrial impairment and cellular oxidative damage, and the cell survival rate was significantly higher than Gly-untreated yeast. The yeast cells exposed to Gly significantly increased the activities of antioxidant enzymes superoxide dismutase (SOD), catalase (CAT), and the content of glutathione (GSH). Notably, Gly-treated yeast cells had better biocontrol efficacy against *A. niger* in postharvest apples. The lesion diameter and decay incidence were reduced by 17.67 mm and 79.63% compared to the control, respectively, when *S. pararoseus* was treated with 1 mM Gly. Moreover, Gly-treated yeast increased the antioxidant enzymes activities and their gene expression were up-regulated in apples. These results indicated that 1 mM Gly not only reduced the oxidative damage of yeast, but also induced resistance-related enzymes of apples under oxidative stress, which contributed to enhancing the biocontrol efficacy of *S. pararoseus* against *A. niger* in apples.

## 1. Introduction

Apple is a globally popular fruit that is enriched with vitamins, citric acid, malic acid, and other nutrients [1]. However, apples are vulnerable to decay caused by harmful fungi during storage, transportation, and commercialization after harvest [2]. At the same time, due to its high sugar content, high water activity and low pH value, spoilage pathogens easily infect apples through wounds, which seriously affects the storage period of apples [3]. *Aspergillus niger* (*A. niger*), *Penicillium expansum* and *Alternaria alternata* are reported to be the predominant decay organisms in apples [4,5,6]. *A. niger* is a fungus that mainly attacks fruits by producing stratum cuticle, enzymes that break down cell walls, toxins, and detoxifying compounds [7]. It can infect flowering and premature fruits or injured berries, resulting in fruit decay. Consequently, the biological control study of *A. niger* is essential for the postharvest storage of apples. Postharvest biological control of fruits has emerged as a promising and potential method for controlling fruit decay and spoilage in recent years. However, in practical applications, biological control usually cannot achieve the same stable effect as chemical fungicides and part of the reason is because of the various stresses in the environment (such as temperature, pH, UV, osmotic and oxidative stresses) [8].

Yeast cells, considered a promising microbial antagonist, accumulate significant amounts of ROS, causing severe oxidative damage under environmental stress, such as O_2_- and H_2_O_2_ [9]. Moreover, when the antagonistic yeast grows on the fruit, it not only accumulates more ROS, but also induces the fruit and pathogenic fungi to generate a large amount of ROS instantaneously in the wound, consequently leading to oxidation explosion in the wound [10]. Intense oxidative stress can damage DNA, lipids, proteins, and other cellular components, seriously reducing the biological and biocontrol efficacy of antagonistic yeast [11,12]. Therefore, improving the tolerance of antagonistic yeast to oxidative stress is of great significance in practical biocontrol applications.

Gly plays a crucial role in regulating key biological processes for life, growth, nutritional utilization, immunity, and reproduction. It was reported that Gly is widely used as an osmotic protectant that significantly improves the high permeability resistance of *Saccharomyces cerevisiae* [13,14]. Furthermore, Gly mitigates the oxidative damage in *Pediococcus pentosaceus*, reducing the levels of malondialdehyde, carbonylated protein, and the generation of intracellular ROS [15]. Gly’s mitigation effect has also been confirmed against oxidative stress in mice, shrimp, and the human body [16,17]. However, there is no research on its effects on the oxidative stress tolerance of yeast and its application in biological control.

The aim of present study is to determine the effect of Gly on oxidative stress tolerance and biocontrol efficacy of *S. pararoseus* CC-HS1 against *A. niger* in apples. Specifically, we investigated the effect of exogenous Gly in *S. pararoseus* CC-HS1 exposed to H_2_O_2_ (i) on ROS accumulation, oxidative damage and antioxidant enzymes; (ii) on antioxidant-related enzymic activities of apples; and (iii) on biocontrol efficacy. The present study suggested a promising method to protect *S. pararoseus* CC-HS1 under oxidative stress and control the decay of apples caused by *A. niger* as well as elucidated the mechanism.

## 2. Materials and Methods

### 2.1. Strains

*Sporidiobolus pararoseus* (*S. pararoseus*) CC-HS1 was isolated from the surface of a healthy cherry tomato as described in our previous article [18]. The yeasts were identified by constructing a phylogenetic tree in ITS domain sequences (Figure S1) and stored with 20% glycerol at −80 °C until use. Then, *S. pararoseus* CC-HS1 was revived in YPD liquid medium (20 g peptone, 20 g dextrose, and 10 g yeast extract in 1 L water) at 30 °C, 120 rpm on a rotary shaker for 36 h. The yeast cells were centrifuged at 8000× *g* for 5 min and washed three times with sterile distilled water (SDW). Then, the cell was resuspended in SDW and adjusted to the required concentration for the following experiments.

*Aspergillus niger* (*A. niger*, CICC-2487) was obtained from the China Center of Industrial Culture Collection (CICC), Beijing, China. It was incubated at 28 °C for 7 days on potato dextrose agar (PDA). Spores of the pathogen were eluted using sterile saline that included 0.05% (*v/v*) Tween 80. The spore suspension was then resuspended in SDW and adjusted using a hemocytometer to the concentration of 1 × 10^5^ spores/mL.

### 2.2. Fruits

Apples (cv. ‘Ralls’) were harvested from an orchard after achieving commercial maturity in the Liaoning Province. These apples were selected for uniformity of size and maturity and did not have apparent damage. After being washed twice with SDW and disinfected for 2 min with 0.2% sodium hypochlorite, each fruit sample was air dried at room temperature.

### 2.3. Evaluation of Survival of S. pararoseus CC-HS1 under Oxidative Stress

The oxidative stress tolerance of *S. pararoseus* CC-HS1 exposed to H_2_O_2_ was determined based on the method mentioned by Wang et al. [19]. Equal volumes of H_2_O_2_ solutions at different concentrations of 5, 10, 20, and 40 mM were added to 10 mL of yeast cultures (1 × 10^8^ cells/mL). The mixture was then incubated at 30 °C at 120 rpm for 60 min. Subsequently, centrifugation was performed to obtain the yeast cells. Then, the cells were washed twice using SDW and finally adjusted to a concentration of 2 × 10^7^ cells/mL. Sequential 10-fold dilutions (ranging from 2 × 10^6^ to 2 × 10^3^ cells/mL) of 100 μL samples were then spread on YPD agar plates. The results of counting the yeast colonies were expressed as lg CFU/mL after incubation at 30 °C for 48 h. Moreover, when conducting the yeast spotting assay, we utilized a 5 μL serial 10-fold dilution method for spotting onto YPD agar plates and then cultured at 30 °C.

### 2.4. Determination of Gly Concentration on Oxidative Stress Tolerance of S. pararoseus CC-HS1

The effects of *S. pararoseus* CC-HS1 treated with different concentrations of Gly followed by exposure to H_2_O_2_ were assessed according to a previous study [20]. Yeast cells (1 × 10^8^ cells/mL) were resuspended in 50 mL of fresh YPD culture medium, which was then supplemented with various concentrations of Gly (0.5 mM, 1 mM, 1.5 mM, and 2 mM) and incubated at 30 °C, 120 rpm for 24 h. Yeast cells without Gly and H_2_O_2_ treatment served as the control group. After incubation, centrifugation at 8000× *g* for 5 min was employed to harvest the yeast cells. Subsequently, a triple washing procedure with SDW was conducted on the collected cells. Then, the control, Gly-untreated, and Gly-treated yeast samples with a concentration of 1 × 10^8^ cells/mL underwent exposure to 10 mM H_2_O_2_ at 30 °C, 120 rpm for 60 min, and survival rates were evaluated using the methodology described in Section 2.3.

### 2.5. Effect of Gly on Oxidative Damage of S. pararoseus CC-HS1

#### 2.5.1. Imaging of Intracellular ROS and Mitochondrial Membrane Potential Determination

The intracellular ROS production in *S. pararoseus* CC-HS1 was assessed using the oxidant-sensitive probe, 2,7-dichlorodihydrofluorescein diacetate (DCHF-DA) (Solarbio, Beijing, China), following the method described by Liu et al. [21]. Yeast cells were cultured in YPD liquid medium containing 0 and 1 mM Gly at 30 °C for 24 h, and then adjusted to a concentration of 1 × 10^8^ cells/mL. Then, yeast cells, both Gly-untreated and treated, were exposed to 10 mM H_2_O_2_ for 60 min. *S. pararoseus* CC-HS1 without Gly and H_2_O_2_ treatment served as the control group. Phosphate-buffered saline (PBS) was used to wash the cells twice, which were resuspended at a concentration of 5 × 10^7^ cells/mL. Then, 25 µM DCHF-DA was added to the suspension, which was dissolved in dimethyl sulfoxide, followed by incubation for 1 h at 30 °C in darkness. A fluorescence microscope (Olympus, Tokyo, Japan) fitted with a UV light source, a 485 nm excitation and a 530 nm emission filter combination were used to view the yeast cells. From each slide, we randomly selected five fields of view, which included at least 100 cells. The calculation of ROS levels involved determining the percentage by comparing the number of cells exhibiting fluorescence to the total number of cells captured in the bright field image, multiplied by 100.

The mitochondrial membrane potential (ΔΨm) of the yeast cells (Gly-treated and untreated) exposed to 10 mM H_2_O_2_ for 60 min (1 × 10^8^ cells/mL) was measured; refer to Nie et al. [22]. The ΔΨm, indicative of cellular damage, was quantified using a kit that contained the cationic dye JC-10 (Solarbio, China). In healthy cells, JC-10 appears as a red fluorescence within the mitochondria, while in compromised cells, the dye appears as a green fluorescence. Yeast cells were resuspended in the JC-10 reagent (1 × 10^6^ cells/mL). After incubation for 20 min at 37 °C, they were collected and a fluorescent microplate reader (Spank, Tecan, Männedorf, Switzerland) was employed to determine the fluorescence ratio of red to green.

#### 2.5.2. Assay of Membrane Integrity and Apoptosis

Membrane integrity and apoptosis of *S. pararoseus* CC-HS1 cells were assessed based on the method of He et al. [23]. Yeast cells, both Gly-untreated and treated, were harvested as described in Section 2.5.1 (1 × 10^8^ cells/mL). The cells without Gly and H_2_O_2_ treatment served as control. The yeast cells were collected via centrifuging and washing twice in PBS, and then they were re-suspended in a comparable volume of PBS containing 10 μg/mL propidium iodide (PI) and 5 μg/mL Hoechst 33342. Subsequently, the yeast cells were incubated at room temperature in darkness for 20 min, followed by washing twice. The yeast cells were observed with a fluorescence microscope (Olympus, Tokyo, Japan). PI is a membrane-impermeable fluorescent dye; cells with compromised membrane integrity appear red. Apoptotic cells were presented as Hoechst 33342-positive and PI-negative; each field of view had at least 100 cells, and the percentage of apoptotic cells and the integrity of the plasma membrane were determined, respectively.

#### 2.5.3. Measurement of Lipid Peroxidation and Antioxidant Enzymes Activities

The malonaldehyde (MDA) levels were measured using an MDA assay kit (Solarbio, Beijing, China), following the manufacturer’s instructions [24]. *S. pararoseus* CC-HS1 cells (Gly-treated and untreated) were harvested as described in Section 2.5.1 (1 × 10^8^ cells/mL). Yeast cells were collected via centrifugation and then washed twice with SDW. These yeast cell samples were broken using glass beads, resuspended in the extraction solution, and centrifuged at 8000× *g*, 4 °C for 5 min. The subsequent experiments made use of the resultant supernatant. The MDA content was expressed as mmol/kg protein.

Yeast cells (both Gly-untreated and treated) were harvested as described in Section 2.5.1 (1 × 10^8^ cells/mL). The yeast cells without Gly and H_2_O_2_ treatment served as control. Yeast cells were disrupted via glass beads and suspended in their respective extraction solutions. After centrifugation at 8000× *g* and at 4 °C for 5 min, the supernatant from the combination was utilized for enzyme assays. The catalase (CAT) and superoxide dismutase (SOD) activities were measured using CAT and SOD assay kits (Solarbio, Beijing, China) at absorbances of 560 nm and 240 nm, respectively. The content of glutathione (GSH) was measured using GSH assay kits (Solarbio, Beijing, China) at 412 nm absorbance and expressed as ug/mg protein. Both the SOD and CAT activities were represented as U/mg protein.

### 2.6. Assay of S. pararoseus CC-HS1 Treated with Gly on Defense-Related Enzyme Activities in Apples

Yeast cells, both Gly-treated and untreated, were harvested as described in Section 2.5.1 (1 × 10^8^ cells/mL). Three uniform wounds (approximately 3 mm deep × 5 mm wide) were made at the equator of the apple samples using a sterile needle [25]. A total of 10 μL of SDW (control), yeast cells exposed to H_2_O_2_ (1 × 10^8^ cells/mL), and yeast cells treated with Gly followed by exposure to H_2_O_2_ (1 × 10^8^ cells/mL) was added to each respective wound. The apples were stored at 25 °C for 6 days. The apple wounds were excised using a sterile knife, added to a sterilized pre-cooled mortar, and then ground with a certain amount of extraction buffer. The mixture was centrifuged at 10,000× *g*, at 4 °C for 30 min. The defense-related enzyme activities were determined by assaying the supernatant. The enzyme activities were expressed as U/g fresh weight (FW).

The activities of polyphenol oxidase (PPO) and peroxidase (POD) were measured [26]. Briefly, 1 g of apple tissue was homogenized and combined with 5 mL of extraction buffer containing 4% PVPP and 1.33 mM EDTA. The rise in absorbance of 0.01, at 398 nm and 470 nm, per minute per gram of apple tissue, was used to define the enzyme activities of PPO and POD, respectively.

Phenylalanine aminolytic (PAL) enzymatic activity was determined according to the method of Li et al. [27]. A total of 5 mL of extraction buffer containing 40 g/L PVP, 2 mmol/L EDTA, and 5 mmol/L β-mercaptoethanol was added to 1 g of apple pulp. The absorbance was measured at 290 nm. An enzyme activity unit was defined as a 0.01 change in absorbance per hour.

The CAT activity was determined using the method of Lai et al. [28], with slight modifications. One gram of apple pulp was grounded in a mortar, and five mL of pH 7.8 phosphate buffer was pre-cooled at 4 °C. The supernatant (0.2 mL) was added to 2.8 mL of 30 mM H_2_O_2_ and was then measured at the absorbance of 240 nm after 10 s. The change in CAT activity at 240 nm was measured once every 1 min for 3 min. The results were calculated by increasing the absorbance value by 0.01 to determine 1 unit of enzyme activity. 

### 2.7. Assay of S. pararoseus CC-HS1 Treated with Gly on the Expression Levels of Defense-Related Genes Expression in Apples (RT-qPCR)

The apples were prepared and grouped following the description in Section 2.6. Total RNA collected from apple pulp was isolated with an RNAprep Pure Plant kit (Tiangen, Beijing, China), and the cDNA was synthesized using the ABM OneScript^®^ Plus cDNA synthesis Kit. The primers designed using Primer3 are shown in Table 1. Then, qPCR was measured via SYBR BlasTaq 2X qPCR MasterMix on the PCR system (Bio-Rad CFX96Touch, Hercules, CA, USA). Real-time PCR was performed to analyze the relative expression levels of genes, and the data were normalized using Ct values corresponding to ACTIN. The gene expression levels were calculated using the 2^−ΔΔCT^ method.

### 2.8. Population Dynamics and Biocontrol Assay of Gly-Treated S. pararoseus CC-HS1 in Apple Wounds

The apples were prepared and grouped following the description in Section 2.6 and incubated at 25 °C. Samples were collected for six consecutive days for counting. The other samples cultivated at 4 °C were collected at 3-day intervals for a duration of 21 days. All of the samples collected at 2 h after incubation served as day 0. The samples were removed from wounds as 10 mm × 10 mm with a sterile knife and were mixed with 3 mL of SDW and homogenized. Then, 200 μL of the homogenate was serially diluted and spread onto YPD agar medium, followed by incubation at 30 °C for 36 h. The colonies of *S. pararoseus* were enumerated, and the results were quantified in terms of lg CFU/wound [29].

The biocontrol ability of Gly-treated *S. pararoseus* CC-HS1 was evaluated on apples [30]. Apples were prepared as described in Section 2.6. *S. pararoseus* CC-HS1 was cultured in YPD media containing different concentrations of Gly (0, 0.5, 1, 1.5, and 2 mM) at 30 °C for 24 h. Then, the yeast cells were exposed to 10 mM H_2_O_2_ for 60 min. Afterward, the apples were randomly divided into 7 groups, and each apple wound was inoculated with 10 μL of (A) *S. pararoseus* (*S. p*), (B) control group (SDW), (C) *S. pararoseus* exposed to H_2_O_2_ (*S p* + H_2_O_2_), (D) 0.5 mM Gly-treated *S. pararoseus* followed by exposure to H_2_O_2_ (*S.p* + 0.5 mM Gly + H_2_O_2_), (E) 1 mM Gly-treated *S. pararoseus* followed by exposure to H_2_O_2_ (*S p* + 1 mM Gly + H_2_O_2_), (F) 1.5 mM Gly-treated *S. pararoseus* followed by exposure to H_2_O_2_ (*S.p* + 1.5 mM Gly + H_2_O_2_), and (G) 2 mM Gly-treated *S. pararoseus* followed by exposure to H_2_O_2_ (*S p* + 2 mM Gly + H_2_O_2_). After 2 h, 10 μL of a *A. niger* spore suspension (1 × 10^5^ spores/mL) was piped to each wound of each treatment group. The treated apples were then wrapped with a plastic wrap and stored at 25 °C. Then, the decay incidence (DI) and the lesion diameter (LD) on each fruit was recorded after 5 days.

### 2.9. Statistical Analysis

All of the experiments were performed in triplicate. The data of all experiments were analyzed with SPSS version 26 software, and the error bars indicate the standard deviation (SD) of three replications. All of the data were subjected to analysis of variance (ANOVA) followed by Duncan’s multiple range tests, and *p* < 0.05 was considered significantly different.

## 3. Results

### 3.1. Survival of S. pararoseus CC-HS1 under H_2_O_2_ Oxidative Stress

**Figure 1:**
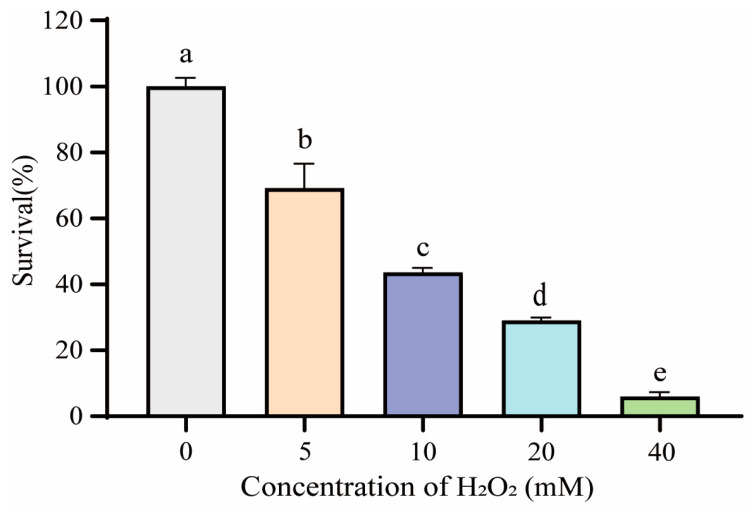
Viability of *S. pararoseus* exposed to various concentrations of H_2_O_2_ for 60 min. The different lowercase letters indicate the significant differences (*p* < 0.05).

As expected, the oxidative stress induced by H_2_O_2_ lead to a concentration-dependent decrease in the cell viability of *S. pararoseus* CC-HS1. The cell viability gradually decreased as the concentration of H_2_O_2_ increased from 0 to 40 mM within a duration of 60 min, as depicted in Figure 1. The 10 mM H_2_O_2_ was moderately lethal to the cells (about 50% inhibitory), which was selected as the appropriate oxidative stress to *S. pararoseus* CC-HS1 in the subsequent studies.

### 3.2. Effect of Gly on Oxidative Stress Tolerance of S. pararoseus CC-HS1

**Figure 2:**
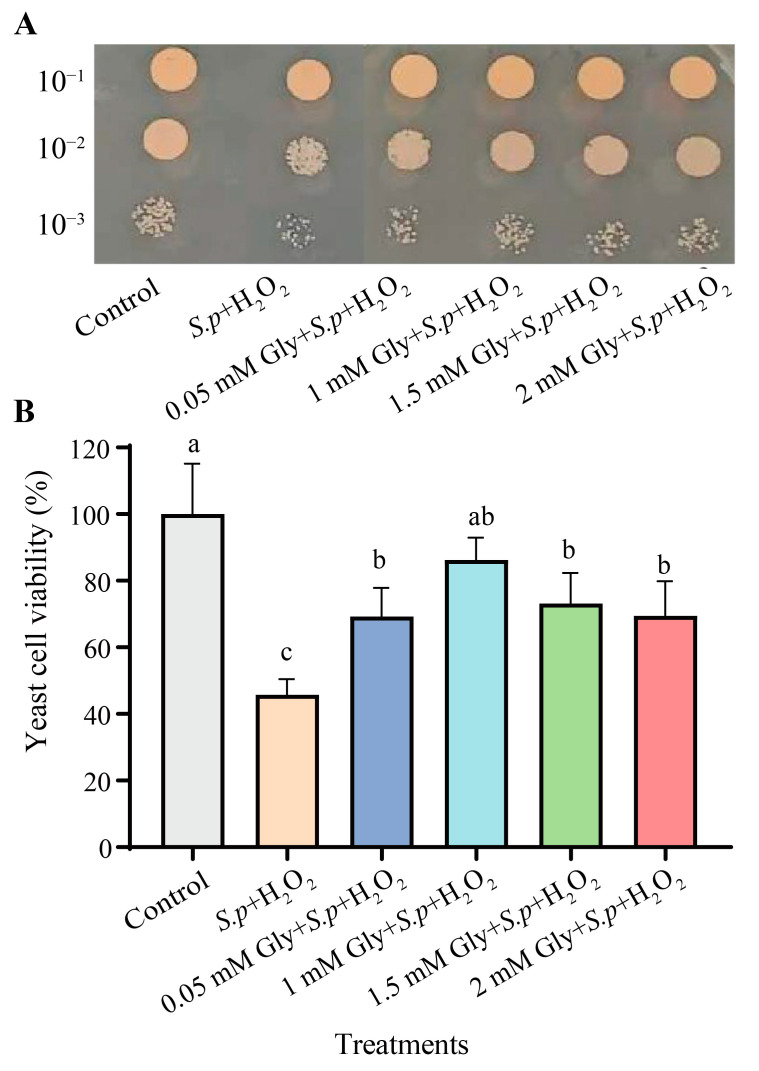
In vitro viability of *S. pararoseus* CC-HS1 from different treatments under 10 mM H_2_O_2_. Images of yeast spotting assays for determining the viability of *S. pararoseus* CC-HS1 (**A**). Viability of yeast cells after different treatments (**B**). Control: *S. pararoseus* CC-HS1; *S.p* + H_2_O_2_: *S. pararoseus* CC-HS1 exposed to H_2_O_2_; 0.05 mM + *S.p* + H_2_O_2_: *S. pararoseus* CC-HS1 treated with 0.05 mM Gly followed by exposure to H_2_O_2_; 0.05 mM + *S.p* + H_2_O_2_: *S. pararoseus* CC-HS1 treated with 0.05 mM Gly followed by exposure to H_2_O_2_; 1.5 mM + *S.p* + H_2_O_2_: *S. pararoseus* CC-HS1 treated with 1.5 mM Gly followed by exposure to H_2_O_2_; 2 mM + *S.p* + H_2_O_2_: *S. pararoseus* CC-HS1 treated with 2 mM Gly followed by exposure to H_2_O_2_. The different lowercase letters indicate the significant differences (*p* < 0.05).

The effect of Gly on the enhancement of yeast cell viability can be observed in Figure 2. The viability of Gly-treated yeast cells was significantly higher than that of untreated cells under oxidative stress (*p* < 0.05). Yeast viability exhibited a significant increase by 40.53% in the 1 mM Gly + *S.p* + H_2_O_2_ group compared to the *S.p* + H_2_O_2_ group. The results demonstrate that the viability of *S. pararoseus* CC-HS1 treated with 1 mM Gly was the best under oxidative stress.

### 3.3. Evaluation of S. pararoseus CC-HS1 Oxidative Damage under Oxidative Stress

#### 3.3.1. ROS Accumulation and Mitochondrial Membrane Potential of *S. pararoseus* CC-HS1

**Figure 3:**
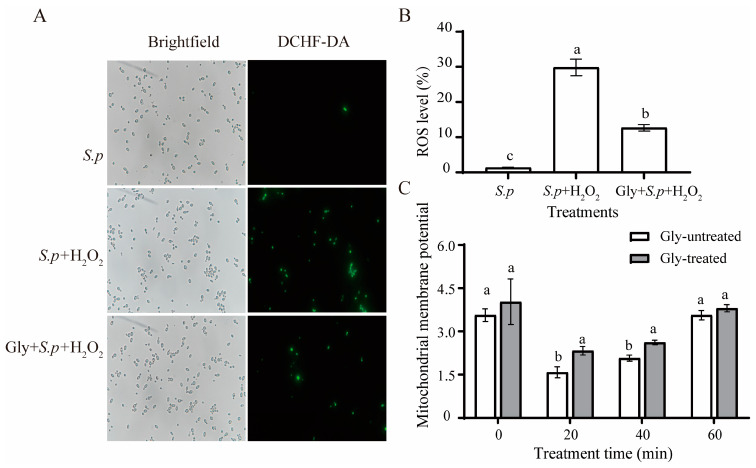
Intracellular ROS levels in *S. pararoseus* CC-HS1 under oxidative stress. Fluorescence microscopic images of *S. pararoseus* CC-HS1 cells stained with the fluoroprobe DCHF-DA under brightfield and UV light (**A**). Percentages of *S. pararoseus* CC-HS1 cells exhibiting visible ROS accumulation (**B**). Mitochondrial membrane potentials of *S. pararoseus* CC-HS1 following treatment with 1 mM Gly under oxidative stress (**C**). *S.p*.: *S. pararoseus* CC-HS1; *S.p*. + H_2_O_2_: *S. pararoseus* CC-HS1 exposed to H_2_O_2_; *S.p*. + Gly + H_2_O_2_: *S. pararoseus* CC-HS1 treated with 1 mM Gly followed by exposure to H_2_O_2_. The different lowercase letters indicate significant differences (*p* < 0.05).

As illustrated in Figure 3, the percentage of ROS-positive yeast cells is 1.3% in *S. pararoseus* group. However, the ROS percentages of Gly-untreated and Gly-treated cells are 29.83% and 12.67% (*p* < 0.05), respectively. These results showed that a large amount accumulation of intracellular ROS can be promoted in *S. pararoseus* CC-HS1 under oxidative stress, but Gly can effectively prevent it in yeast cells.

To investigate whether changes in ROS are related to mitochondrial damage, the mitochondrial membrane potential (ΔΨm) was analyzed. Figure 3C demonstrates that when yeasts were exposed to H_2_O_2_ for 20 min, the mitochondrial membrane potential is at its lowest. The Gly-treated group displayed a significantly *(p* < 0.05) higher mitochondrial membrane potential than the Gly-untreated group.

#### 3.3.2. Membrane Integrity and Apoptosis of *S. pararoseus* CC-HS1 under oxidative stress

**Figure 4:**
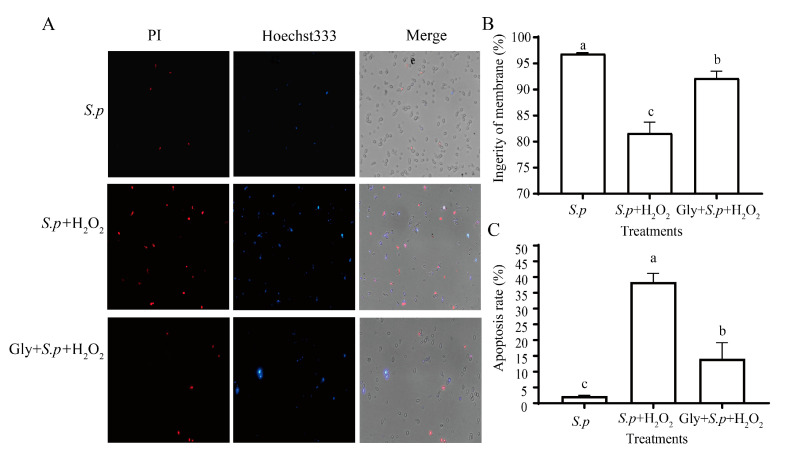
Effects of Gly-treated *S. pararoseus* CC-HS1 on membrane integrity and apoptosis analysis under oxidative stress. Fluorescence microscopic images of *S. pararoseus* CC-HS1 cells double stained with PI/Hoechst 33342 (**A**). Percentages of plasma membrane integrity of *S. pararoseus* CC-HS1 cells (**B**). Apoptosis rate of *S. pararoseus* CC-HS1 cells (**C**). *S.p*.: *S. pararoseus* CC-HS1; *S.p*. + H_2_O_2_: *S. pararoseus* CC-HS1 exposed to H_2_O_2_; *S.p*. + Gly + H_2_O_2_: *S. pararoseus* CC-HS1 treated with 1 mM Gly followed by exposure to H_2_O_2_. The different lowercase letters indicate significant differences (*p* < 0.05).

The apoptosis of *S. pararoseus* CC-HS1 cells was demonstrated with Hoechst 33342 and PI staining (Figure 4A). The integrity of the plasma membrane of *S. pararoseus* CC-HS1 cells exposed to 10 mM H_2_O_2_ was reduced to 81.63%. Additionally, the number of apoptotic yeast cells showed an increase, reaching 38.43% (*p* < 0.05). However, in the *S. p*. group, the plasma membrane damage and apoptosis were not significant (Figure 4B,C). The plasma membrane integrity was increased with Gly treatment to 92.17% and the apoptosis decreased to 14.1%, which is comparable to *S. pararoseus* CC-HS1.

#### 3.3.3. The Lipid Peroxidation and Antioxidant Activities of *S. pararoseus* CC-HS1

**Figure 5:**
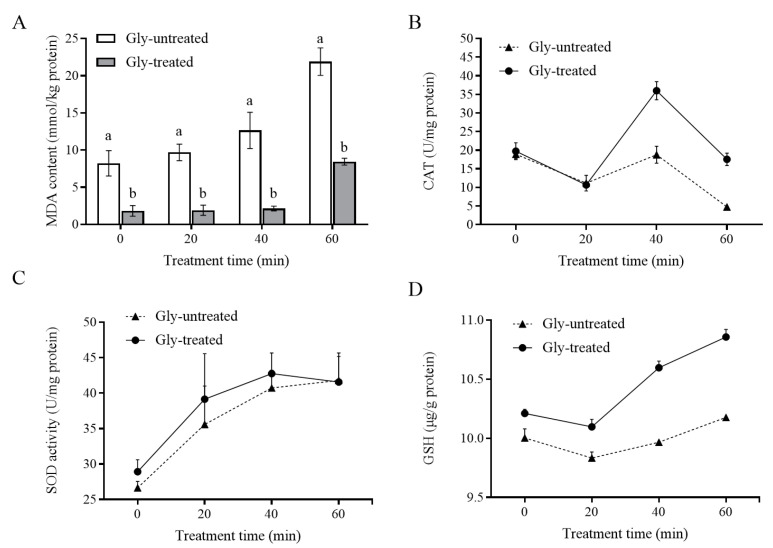
Effects of Gly-treated *S. pararoseus* CC-HS1 exposed to H_2_O_2_ on MDA content (**A**), catalase (CAT) activity (**B**), superoxide dismutase (SOD) activity (**C**), and glutathione (GSH) content (**D**) under oxidative stress. Gly-untreated: yeast cells exposed to H_2_O_2_; Gly-treated: yeast cells treated with Gly followed by exposure to H_2_O_2_. The different lowercase letters indicate significant differences (*p* < 0.05).

The lipid peroxidation (MDA content), antioxidant enzymes activities, and GSH content of *S. pararoseus* CC-HS1 were determined under oxidative stress. The content of intracellular MDA in Gly-untreated group increased rapidly with the increase in oxidative stress time (Figure 5A). At time 0, the MDA contents in both Gly-untreated and Gly-treated groups were at low levels and were not significantly different between the two groups. Importantly, Gly-treated cells exhibited lower MDA accumulation compared to Gly-untreated cells within 60 min.

In the enzymatic antioxidant defense system, CAT and SOD are two key antioxidant enzymes. The activities of these antioxidant enzymes were evaluated in *S. pararoseus* CC-HS1 exposed to 10 mM H_2_O_2_ for 60 min. Following exposure to 10 mM H_2_O_2_ for 20 min, the Gly-untreated yeast cells only showed a slight decrease in CAT activity within the first 20 min, which was similar to the Gly-untreated group. However, following 40 min of exposure to H_2_O_2_, Gly-treated yeast cells caused a nearly twofold increase in CAT activity compared to untreated yeast cells (Figure 5B). Moreover, the activity of SOD in Gly-treated yeast cells remained at a higher level compared to the untreated yeast cells when they were exposed to H_2_O_2_ from 0 to 60 min (Figure 5C). These suggested that under oxidative stress, the Gly treatment increased SOD activity of *S. pararoseus* CC-HS1 during 40 min, and increased CAT activity for the entire treatment time.

Glutathione is an important antioxidant and a free radical scavenger. After incubation for 60 min, the Gly-treated cells showed a higher GSH content (10.86 µg/g) than the untreated cells (10.18 µg/g).

### 3.4. Effect of Gly-Treated S. pararoseus CC-HS1 on Defense-Related Enzyme Activity in Apples

**Figure 6:**
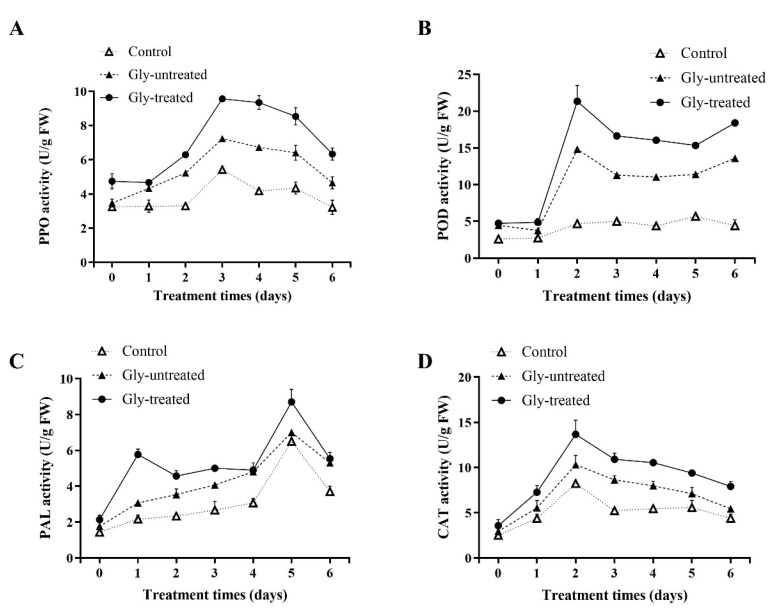
Effects of *S. pararoseus* CC-HS1 treated with Gly on the activities of polyphenol oxidase (PPO) (**A**), peroxidase (POD) (**B**), phenylalanine aminolytic (PAL) (**C**), and CAT (**D**). Control: sterile distilled water; Gly-untreated: yeast cells exposed to H_2_O_2_; Gly-treated: yeast cells treated with Gly followed by exposure to H_2_O_2_. The different lowercase letters indicate significant differences (*p* < 0.05).

To assess the induced resistance of apples treated with *S. pararoseus* CC-HS1 that were Gly-treated, the activities of apple defense-related enzymes were measured. Both the Gly-treated and untreated yeast increased the PPO activity in apples, but the Gly-treated yeast was better (Figure 6A). In the Gly-treated group, the PPO activity reached a peak at 3 days, 6.29 U/g FW, which was 1.76 times higher than that of the control group. Furthermore, the activity of PPO treated with Gly was higher than the untreated group. Likewise, the POD activity in apples exhibited significant changes over 6 days in all of the treatment groups (Figure 6B); the peak value was 21.33 U/g at 2 days, compared to the Gly-untreated group’s peak value of 14.81 U/g. It was indicated that the PAL activity in the Gly-treated group demonstrated a fluctuating upward trend (Figure 6C). The PAL activity in the control, Gly-untreated, and Gly-treated groups reached their peak at 5 days, with a value of 6.5 U/g FW, 7 U/g FW, and 8.7 U/g FW, respectively. These findings indicated that the treatment of Gly had a positive effect on the PAL activity of apples during 6 days. The CAT activity of the control, Gly-untreated and Gly-treated groups reached their maximum activity at 2 days (8.23 U/g FW, 10.3 U/g FW, and 13.67 U/g FW, respectively). These results indicated that the treatment of yeast and Gly could enhanced CAT activity in apples.

### 3.5. Effect of S. pararoseus CC-HS1 Treated with Gly on Defense-Related Gene Expression in Apples

**Figure 7:**
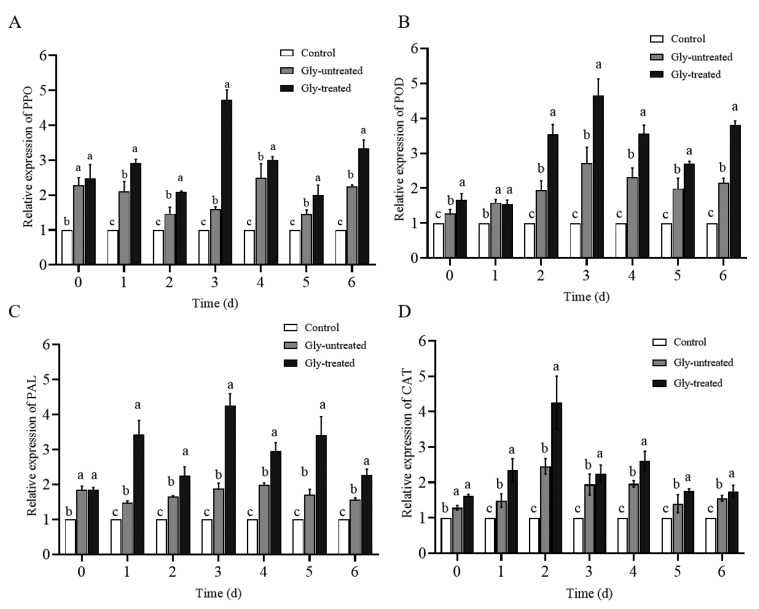
Effects of *S. pararoseus* treated with Gly on relative expression of PPO (**A**), POD (**B**), PAL (**C**), and CAT (**D**). Control: sterile distilled water; Gly-untreated: yeast cells exposed to H_2_O_2_; Gly-treated: yeast cells treated with Gly followed by exposure to H_2_O_2_. The different letters indicate significant differences (*p* < 0.05).

The expression levels of relative defense-related genes were significantly up-regulated after treating the apples with *S. pararoseus* CC-HS1 and Gly-treated *S. pararoseus* CC-HS1 in apples (Figure 7). In our study, the relative gene expression levels of PPO, POD, PAL, and CAT were increased with the Gly treatment, in line with their defense-related enzyme activities. The relative expression levels of PPO, POD, and PAL reached their peak at 3 days (4.73-, 4.66-, and 4.26-fold, respectively), while the CAT in Gly-treated apples peaked at 2 days, a 4.25-fold increase compared to the control group.

### 3.6. Population Dynamics and Biocontrol Assay of Gly-Treated S. pararoseus CC-HS1 in Apple Wounds

*S. pararoseus* CC-HS1 multiplied rapidly in apple wounds at both 25 °C and 4 °C. Moreover, the yeast population treated with Gly was higher than that of the Gly-untreated yeast. The population of Gly-treated yeast cells increased sharply from 6.53 CFU/wound to 6.75 CFU/wound within the first day at 25 °C. The population of Gly-treated and Gly-untreated yeast reached their maximum at 3 days (6.83 and 6.98 CFU/wound, respectively) when they were incubated at 25 °C. The number of yeast cells in the apple wounds increased during 6 days at 25 °C, and similarly during 21 days at 4 °C. The population of Gly-treated cells increased from 6.28 CFU/wound at 0 days to 6.68 CFU/wound at 21 days. Furthermore, the maximum population was observed at 9 days in both the Gly-untreated and Gly-treated groups, reaching 6.67 CFU/wound and 6.87 CFU/wound, respectively.

**Figure 8:**
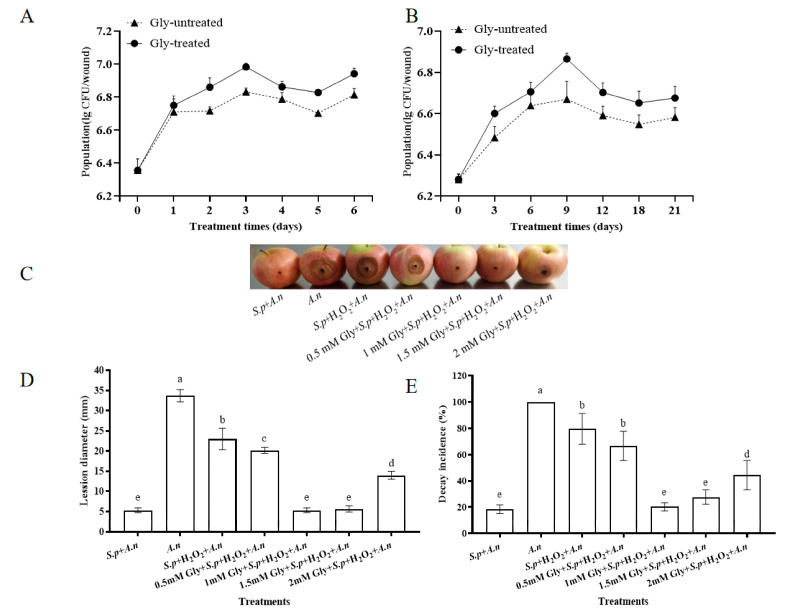
Population dynamics of 1 mM Gly-treated *S. pararoseus* CC-HS1 in wounds at 25 °C (**A**) and 4 °C (**B**). Symptoms of *A. niger* in apple stored at 25 for 5 days after inoculation (**C**). Induction of various concentrations of Gly in *S. pararoseus* CC-HS1 on the lesion diameter (**D**) and decay incidence (**E**) of postharvest *Aspergillus* rot in apples. *S.p + A.n*: *S. pararoseus* CC-HS1 + *A. niger*; *A.n: A. niger*; *S.p* + H_2_O_2_*+ A.n*: *S. pararoseus* CC-HS1 exposed to H_2_O_2_ + *A. niger*; 0.05 mM + *S.p* + H_2_O_2_ + *A. niger*: *S. pararoseus* CC-HS1 treated with 0.05 mM Gly followed by exposure to H_2_O_2_ + *A. niger*; 0.05 mM + *S.p* + H_2_O_2_ + *A. niger*: *S. pararoseus* CC-HS1 treated with 0.05 mM Gly followed by exposure to H_2_O_2_ + *A. niger*; 1.5 mM + *S.p* + H_2_O_2_ + *A. niger*: *S. pararoseus* CC-HS1 treated with 1.5 mM Gly followed by exposure to H_2_O_2_ + *A. niger*; 2 mM + *S.p* + H_2_O_2_ + *A. niger*: *S. pararoseus* CC-HS1 treated with 2 mM Gly followed by exposure to H_2_O_2_ + *A. niger*. The different letters indicate significant differences (*p* < 0.05).

To explore the effects of Gly-treated *S. pararoseus* on biocontrol efficacy in apples, the yeast treated with Gly was measured (Figure 8C–E). The biocontrol efficacy of *S. pararoseus* was significantly reduced in 10 mM H_2_O_2_ oxidative stress conditions. However, it was improved with Gly treatment at all concentrations during 5 days of storage. Compared to the Gly-untreated group, the lesion diameters were reduced by 2.83 mm, 17.67 mm, 17.33 mm, and 9 mm, respectively, when apples were inoculated with yeast cells treated with various concentrations (0.5 mM, 1 mM, 1.5 mM, and 2 mM) of Gly. In conclusion, *S. pararoseus* treated with 1 mM Gly exhibited the most effective inhibition against *A. niger* disease incidence and the smallest decay diameter in postharvest apples.

## 4. Discussion

Apples are easily infected by spoilage mold, which reduces their edible and commercial values. Therefore, certain measures need to be taken during apple storage to prolong the shelf-life of the fruit. In recent years, there has been an exploration of using biological control agents for environmental protection and human health [31]. Yeasts hold a significant position among the various microbial antagonists due to their environmentally friendliness, biocontrol efficacy against pathogens, and potential for genetic improvement. Antagonistic yeasts are considered as biological control agents due to their excellent characteristics and superiority in application. However, antagonistic yeasts frequently exhibit lower reliability compared to chemical methods, prompting researchers to consider treating yeast with a chemical agent to enhance its biocontrol efficiency. Adding antioxidants is a useful strategy for improving the oxidative stress tolerance [32]. In previous studies, exogenous glutathione reduced the apoptosis of *Cryptococcus laurentii* under oxidative stress and then improved its viability and biocontrol efficiency against *Penicillium* infection of postharvest pear fruit [33]. Gly has been known as a principal antioxidant, maintaining the redox balance of cells [34]. Additionally, Gly has been reported to possess antioxidant properties and enhance stress tolerance in plants [35,36]. In this study, the Gly could improve the oxidative stress tolerance of *S. pararoseus* CC-HS1 and showed better biocontrol efficacy on the postharvest *A. niger* rot of apples.

Large quantities of intracellular ROS are generated when yeasts are exposed to oxidative stress, serving as a key factor influencing both cell viability and biocontrol efficacy [37]. The addition of an appropriate amount of Gly to YPD liquid medium improves the viability of *S. pararoseus* CC-HS1 (Figure 2). We found that Gly decreased the ROS levels of yeast cells under the oxidative stress of 10 mM H_2_O_2_ (Figure 1), compared to those without Gly treatment (Figure 3A,B). These results may be ascribed to the ability of *S. pararoseus* CC-HS1 to detoxify a certain amount of ROS was induced by Gly. Similarly, a previous study showed that ascorbic acid reduced the ROS levels of *Pichia caribbica* under oxidative stress and weakened the oxidative damage to cells [38]. Liu observed that glycine betaine also reduced ROS levels in *C. infirmominiatum* cells to reduce its apoptosis rate [39]. The production of ROS is frequently accompanied by mitochondrial damage, which is characterized by a collapse of the mitochondrial membrane potential [40]. As indicated in Figure 3C, the mitochondrial membrane potential was higher in Gly-treated cells compared to Gly-untreated *S. Pararoseus* CC-HS1. To further verify the oxidative damage occurring when *S. Pararoseus* CC-HS1 was exposed to 10 mM H_2_O_2_, the membrane integrity, apoptosis, and MDA were measured. As can be seen in Figure 4, Gly-treated cells increased the membrane integrity and decreased apoptosis. MDA serves as the ultimate product of lipid peroxidation; a higher MDA content indicates more serious oxidative damage [41]. The data in Figure 5A revealed that Gly-treated cells effectively resisted oxidative stress and significantly reduced the content of MDA within 60 min. These findings are consistent with those of previous studies, indicating that adding β-Glucan could induce *Cryptococcus laurentii* growth and also effectively reduce the content of MDA in fruit to weaken oxidative damage [42]. Therefore, Gly could protect yeast cells from oxidative stress damage by inhibiting the accumulation of ROS in cells.

In order to reduce the excessive accumulation of ROS, yeasts have evolved an antioxidant defense system, including various antioxidant enzymes (CAT, SOD, etc.) and non-enzyme components (such as GSH.) [43]. As illustrated in Figure 5B–D, Gly-treated yeast significantly increased the activities of SOD and CAT, along with an increase in the content of GSH under the oxidative stress of 10 mM H_2_O_2_. The antioxidant enzyme activities and non-antioxidant components of yeast cells treated with ascorbic acid, beta-glucan, trehalose, and chitin were greatly increased [38,44]. These results indicate that the increase in antioxidant enzyme activities and non-enzymatic substances induced by Gly may be the key factors in reducing the content of ROS and improving the levels of antioxidation.

In addition, yeast treated with Gly also enhances the activity of apple resistance-related enzymes, including PPO, POD, PAL, and CAT (Figure 6). PPO, an oxidase enzyme, closely associated with the browning of fruit tissue and the plant’s overall resistance to pathogens [41]. POD plays a crucial role in the decomposition of hydrogen peroxide as well as the oxidation of various organic and inorganic compounds involved in plant lignification and ethylene biosynthesis processes. Moreover, both PPO and POD enzymes play a crucial role in synthesizing certain hormones, which ultimately enhances the host’s ability to resist diseases. PAL plays an important role in phenylpropane metabolism, contributing to the synthesis of phytoalexins, lignin, and phenolic substances. While phytoalexins and lignin amplify plant resistance, phenolic substances bolster antioxidant and antibacterial properties. CAT scavenges free radicals, thereby reducing ROS damage. Similar results have been reported by Boen et.al. [45], who investigated the impact of *W. anomalus* on tomatoes resistance-related enzymes. They discovered that the activities of PPO, POD, and SOD in tomatoes were significantly increased by *W. anomalus*. Yan et al. reported that the activities of CAT, POD, and PAL in pears were enhanced via treatment with *M. guilliermondii* [46].

Furthermore, RT-qPCR was used to validate the expressions of the resistance-related enzyme genes in both Gly-untreated and Gly-treated *S. pararoseus* CC-HS1 in apples tissues. During storage, apples inoculated with Gly-treated *S. pararoseus* CC-HS1 increased the expression levels of defense-related genes compared to the control and untreated groups. These findings demonstrate that *S. pararoseus*, whether treated with Gly (1 mM) or not, activates the defense mechanism of apples, and up-regulates the expression of defense genes, thereby enhancing the resistance of apples to pathogens.

Notably, Gly-treated *S. pararoseus* CC-HS1 was able to reduce the rotting rate and lesion diameters of apples more effectively than that of Gly-untreated *S. pararoseus* CC-HS1. This indicates that Gly may affect *A. niger* directly, potentially through the promotion of yeast activities. Yeast growth and reproduction ability are directly related to biocontrol efficacy. Gly positively influenced the growth of *S. pararoseus* CC-HS1. Cell viability directly influences the population dynamics in wound biocontrol, and high population density enables biocontrol to better compete with pathogens for nutrients and space in wounds.

## 5. Conclusions

In summary, Gly can enhance the oxidative stress tolerance of *S. pararoseus* CC-HS1 and improve its biocontrol efficiency against *A. niger*. An optimal concentration of 1 mM Gly significantly increased yeast oxidative stress tolerance and antioxidant activities. Gly-induced *S. pararoseus* CC-HS1 improved the activities of related enzymes (PPO, POD, PAL, and CAT) and the expression levels of genes in apples. The mechanism is related to reducing ROS accumulation, mitigating lipid oxidation, protecting mitochondrial function and enhancing the cell viability of yeast. The application of Gly-treated *S. pararoseus* CC-HS1 is considered as a promising strategy, with practical implications for controlling postharvest apple rot. Further investigations are necessary to explore the nutrition and taste of apples when they are treated with antagonistic yeast. And the yeast components that effectively inhibit *A. niger* are needed to be investigated.

## Figures and Tables

**Table 1:** Primers used in the RT-qPCR analysis of gene expression.

Gene	NCBI Accession No.	Primer Sequence	Annealing Temp. (°C)
PPO	NM_001319261.1	F:aacacccagcccaactttga	60
		R:caaagcttccggcaaactcc	
POD	XM_029098281.1	F: ctgactcggactggttggac	60
		R:agctgagccaaggaatgtcc	
CAT	AY507670.1	F:ggacttcttctcacaccatcca	59.7
		R:gccttgtcgatcagagtgtagg	
PAL	AF494403.1	F:gagccaagtcgcaaagagga	59.5
		R: gcattcttctcactctcgcc	
ACTIN	XM_008393049.3	F: cctccctcatgccatccttc	60
		R: tgactcgtcgtactcaccct	

## Data Availability

Data are contained within the article.

## Supplementary Materials

The following supporting information can be downloaded at: https://www.mdpi.com/article/10.3390/foods12224121/s1, Figure S1: Phylogenetic analysis of CC-HS1 based on the D1/D2 domain of the 26S rDNA (A). Phylogenetic analysis of CC-HS1 based on the ITS region (B). The phylogenetic tree was constructed using MEGA X.
