# Supplementary material for: Glycine Enhances Oxidative Stress Tolerance and Biocontrol Efficacy of Sporidiobolus pararoseus against Aspergillus niger Decay of Apples
Source: Foods. 2023 Nov 14;12(22):4121. doi: 10.3390/foods12224121 (PMC10670768; doi:10.3390/foods12224121)
Supplement: Supplementary file 1 [file foods-12-04121-s001.zip › foods-2667156-supplementary.pdf]

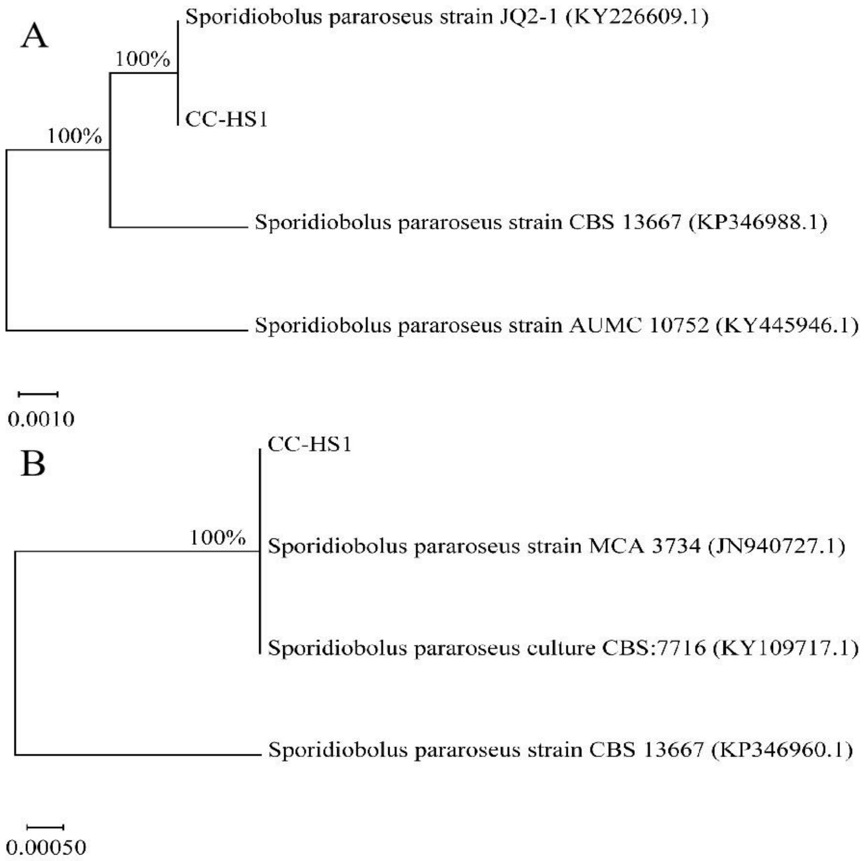

Figure S1. Phylogenetic analysis of CC-HS1 based on the D1/D2 domain of the 26S rDNA (A). Phylogenetic analysis of CC-HS1 based on the ITS region (B). The phylogenetic tree was constructed using MEGA X.
